# Supplementary material for: Genome analysis of Legionella pneumophila ST23 from various countries reveals highly similar strains
Source: Life Sci Alliance. 2022 Mar 2;5(6):e202101117. doi: 10.26508/lsa.202101117 (PMC8899845; doi:10.26508/lsa.202101117)
Supplement: Supplementary file 1 [file LSA-2021-01117_TableS1.docx]

**Table S1**. cgMLST targets differing between ST23 and the ST2695 variant identified in Bresso outbreak in 2018

| **Target** | **Begin** | **End** | **Gene name** | **GenBank**  **protein_ID** | **cgMLST allele number ST2695** | **cgMLST allele number ST23** |
| --- | --- | --- | --- | --- | --- | --- |
| Differing targets in 22,5Kb recombination region | | | | | | |
| lpg0687 | 739735 | 740025 | groES | YP_094723.1 | 2 | 1 |
| lpg0688 | 740047 | 741699 | groEL | YP_094724.1 | 38 | 37 |
| lpg0689 | 741822 | 742262 | - | YP_094725.1 | 23 | 3 |
| lpg0691 | 744892 | 746772 | parE | YP_094727.1 | 43 | 7 |
| lpg0692 | 746894 | 748714 | dppF | YP_094728.1 | 49 | 48 |
| lpg0693 | 748803 | 753083 | - | YP_094729.1 | 69 | 7 |
| lpg0694 | 753445 | 755154 | proS | YP_094730.2 | 41 | 5 |
| lpg0697 | 759971 | 762277 | sul1 | YP_094733.1 | 58 | 7 |
| Differing targets in 8,2Kb recombination region | | | | | | |
| lpg0726 | 794785 | 795252 | nrdR | YP_094762.1 | 9 | 5 |
| lpg0730 | 797274 | 798326 | - | YP_094766.1 | 31 | 4 |
| lpg0732 | 799065 | 799703 | - | YP_094768.1 | 28 | 1 |
| lpg0733 | 799869 | 801317 | - | YP_094769.1 | 47 | 46 |
| lpg0734 | 801369 | 802979 | - | YP_094770.1 | 48 | 6 |
| Differing targets in 9,8Kb recombination region | | | | | | |
| lpg0752 | 822797 | 823867 | neuB | YP_094788.1 | 48 | 47 |
| lpg0753 | 823867 | 825000 | - | YP_094789.1 | 44 | 3 |
| lpg0755 | 825609 | 827111 | yvfE | YP_094791.1 | 4 | 1 |
| lpg0759 | 829724 | 831226 | pgi | YP_094795.1 | 45 | 44 |
| lpg0760 | 831339 | 832256 | rfbA | YP_094796.1 | 45 | 44 |
| Differing targets in 3Kb recombination region | | | | | | |
| lpg0784 | 858280 | 859017 | - | YP_094820.1 | 7 | 3 |
| lpg0786 | 860039 | 861340 | mesJ | YP_094822.1 | 59 | 58 |
| Differing targets in 13,7Kb recombination region | | | |  |  |  |
| lpg0878 | 952719 | 953018 | - | YP_094913.1 | 17 | 16 |
| lpg0879 | 953282 | 954424 | - | YP_094914.1 | 43 | 42 |
| lpg0880 | 954486 | 955127 | - | YP_094915.1 | 21 | 4 |
| lpg0882 | 955610 | 956044 | - | YP_094916.1 | 12 | 4 |
| lpg0883 | 956168 | 956614 | - | YP_094917.1 | 18 | 4 |
| lpg0891 | 964156 | 966471 | - | YP_094925.1 | 90 | 5 |
