# Supplementary material for: Genome analysis of Legionella pneumophila ST23 from various countries reveals highly similar strains
Source: Life Sci Alliance. 2022 Mar 2;5(6):e202101117. doi: 10.26508/lsa.202101117 (PMC8899845; doi:10.26508/lsa.202101117)
Supplement: Supplementary file 2 [file LSA-2021-01117_TableS2.docx]

| **Table S2. Loci of difference found in L. pneumophila strains ST2695, isolated during Bresso 2018 outbreak** | | | | | | | | |
| --- | --- | --- | --- | --- | --- | --- | --- | --- |
| **Locus** | **length** | **Nucleotide substitutions** | **Identity (%)** | **Deletions** | **Insertions** | **N. aminoacidic substitutions** | **N. silent nucleotide substitutions** | **N. missense nucleotide substitutions** |
|  | **(bp)** |  |  | **(bp)** | **(bp)** |  |  |  |
| **lpg0687** | 291 | 3 | 99 | - | - | 0 | 3 | 0 |
| **lpg0688** | 1653 | 24 | 99 | - | - | 0 | 24 | 0 |
| **lpg0689** | 445 | 2 | 100 | - | - | 0 | 2 | 0 |
| **lpg0691** | 1883 | 30 | 98 | - | - | 4 | 26 | 4 |
| **lpg0692** | 1811 | 27 | 99 | - | - | 8 | 19 | 8 |
| **lpg0693** | 4285 | 104 | 98 | - | 3 | 48 | 52 | 49 + 3 bp insertion |
| **lpg0694** | 1711 | 69 | 96 | - | - | 12 | 54 | 15 |
| **lpg0697** | 2308 | 31 | 99 | - | - | 1 | 30 | 1 |
| **lpg0726** | 469 | 2 | 100 | - | - | 0 | 2 | 0 |
| **lpg0730** | 1047 | 10 | 99 | - | - | 3 | 7 | 3 |
| **lpg0732** | 639 | 6 | 99 | - | - | 2 | 4 | 2 |
| **lpg0733** | 1449 | 33 | 98 | - | - | 13 | 19 | 14 |
| **lpg0734** | 1612 | 9 | 99 | - | - | 2 | 7 | 2 |
| **lpg0752** | 1070 | 26 | 98 | - | - | 6 | 20 | 6 |
| **lpg0753** | 1134 | 45 | 96 | - | - | 11 | 32 | 13 |
| **lpg0755** | 1504 | 67 | 96 | - | - | 11 | 50 | 17 |
| **lpg0759** | 1485 | 78 | 95 | - | - | 28 | 46 | 32 |
| **lpg0760** | 916 | 18 | 98 | - | - | 0 | 18 | 0 |
| **lpg0784** | 738 | 18 | 98 | - | - | 2 | 16 | 2 |
| **lpg0786** | 1302 | 31 | 98 | - | - | 10 | 21 | 10 |
| **lpg0878** | 276 | 19 | 93 | - | - | 4 | 15 | 4 |
| **lpg0879** | 1143 | 108 | 91 | - | - | 36 | 64 | 44 |
| **lpg0880** | 643 | 8 | 99 | - | - | 0 | 8 | 0 |
| **lpg0882** | 435 | 7 | 98 | - | - | 1 | 6 | 1 |
| **lpg0883** | 447 | 12 | 97 | - | - | 1 | 11 | 1 |
| **lpg0891** | 2316 | 46 | 98 | - | - | 5 | 41 | 5 |
