# Supplementary material for: Genome analysis of Legionella pneumophila ST23 from various countries reveals highly similar strains
Source: Life Sci Alliance. 2022 Mar 2;5(6):e202101117. doi: 10.26508/lsa.202101117 (PMC8899845; doi:10.26508/lsa.202101117)
Supplement: Supplementary file 3 [file LSA-2021-01117_TableS3.docx]

**Table S3**. cgMLST targets differing between ST23 isolated in Cesano Maderno (2008) and Bresso (2018) LD outbreaks

| **Target** | **Begin** | **End** | **Gene name** | **GenBank**  **protein_ID** | | **cgMLST allele number ST2695** | **cgMLST allele number ST23** | |  |
| --- | --- | --- | --- | --- | --- | --- | --- | --- | --- |
| Differing targets in 4,3Kb recombination region | | | | | | | | | |
| lpg0561 | 600719 | 601465 | phaB | YP_094602.1 | 10 | | | 6 | |
| lpg0562 | 601595 | 601993 |  | YP_094603.1 | 9 | | | 2 | |
| lpg0563 | 602138 | 602494 |  | YP_094604.1 | 12 | | | 11 | |
| lpg0564 | 602739 | 603809 |  | YP_094605.1 | 13 | | | 7 | |
| lpg0565 | 603867 | 604484 |  | YP_094606.1 | 22 | | | 21 | |
| lpg0566 | 604481 | 605014 |  | YP_094607.1 | 24 | | | 2 | |
| Differing targets in 4,4Kb recombination region | | | | | | | | | |
| lpg1136 | 1250582 | 1251484 |  | YP_095169.1 | 11 | | | 38 | |
| lpg1137 | 1252018 | 1252986 |  | YP_095170.1 | 34 | | | 35 | |
| lpg1138 | 1253246 | 1254268 |  | YP_095171.1 | 11 | | | 6 | |
| lpg1139 | 1254265 | 1255032 |  | YP_095172.1 | 9 | | | 6 | |
| Differing targets in 13,7Kb recombination region | | | | | | | | | |
| lpg2439 | 2755062 | 2755613 |  | YP_096447.1 | 8 | | | 5 | |
| lpg2442 | 2757333 | 2757743 |  | YP_096450.1 | 9 | | | 5 | |
| lpg2443 | 2757867 | 2758424 |  | YP_096451.1 | 25 | | | 26 | |
| lpg2446 | 2759982 | 2760260 |  | YP_096454.1 | 19 | | | 20 | |
| lpg2450 | 2762115 | 2762843 | - | YP_096457.1 | 7 | | | 5 | |
| lpg2451 | 2762922 | 2763890 | - | YP_096458.1 | 13 | | | 6 | |
| lpg2452 | 2764049 | 2766814 | legA14 | YP_096459.1 | 13 | | | 7 | |
| lpg2453 | 2767580 | 2768029 | - | YP_096460.1 | 9 | | | 5 | |
| lpg2454 | 2768334 | 2768780 | - | YP_096461.1 | 19 | | | 5 | |
| Differing targets in 8,6Kb recombination region | | | | | | | | | |
| lpg2529 | 2859811 | 2861529 | - | YP_096536.1 | 13 | | | 14 | |
| lpg2530 | 2862296 | 2863339 | aroF | YP_096537.1 | 8 | | | 5 | |
| lpg2534 | 2866611 | 2867042 | - | YP_096541.1 | 5 | | | 2 | |
| lpg2535 | 2867089 | 2867496 | - | YP_096542.1 | 22 | | | 23 | |
