# Supplementary material for: Genome analysis of Legionella pneumophila ST23 from various countries reveals highly similar strains
Source: Life Sci Alliance. 2022 Mar 2;5(6):e202101117. doi: 10.26508/lsa.202101117 (PMC8899845; doi:10.26508/lsa.202101117)
Supplement: Supplementary file 4 [file LSA-2021-01117_TableS4.docx]

| **Table S4. Loci of difference found in L. pneumophila isolates during Cesano Maderno outbreak** | | | | | | | | |
| --- | --- | --- | --- | --- | --- | --- | --- | --- |
| **Locus** | **Length** | **N. nucleotide substitutions** | **Identity (%)** | **Deletions**  **(N. bp)** | **Insertions (N. bp)** | **N. aminoacidic substitutions** | **N. silent nucleotide substitutions** | **N. missense nucleotide substitutions** |
|  | **(bp)** |  |  |  |  |  |  |  |
| **lpg0561** | 747 | 12 | 98 | - | - | 1 | 11 | 1 |
| **lpg0562** | 399 | 7 | 98 | - | - | 1 | 6 | 1 |
| **lpg0563** | 358 | 7 | 97 | 1 | - | STOP codon |  |  |
| **lpg0564** | 1073 | 21 | 98 | - | - | 3 | 17 | 4 |
| **lpg0565** | 530 | 10 | 98 | - | - | 1 | 9 | 1 |
| **lpg0566** | 621 | 7 | 98 | - | - | 0 | 7 | 0 |
| **lpg1136** | 903 | 4 | 99 | - | - | 2 | 2 | 2 |
| **lpg1137** | 975 | 29 | 97 | - | 6 | 12 | 14 | 9 + 6 bp insertion |
| **lpg1138** | 763 | 16 | 97 | - | - | 1 | 14 | 2 |
| **lpg1139** | 1023 | 13 | 98 | - | - | 4 | 9 | 4 |
| **lpg2439** | 552 | 13 | 97 | - | - | 2 | 11 | 2 |
| **lpg2442** | 411 | 7 | 98 | - | - | 2 | 5 | 2 |
| **lpg2443** | 558 | 7 | 98 | - | - | 1 | 6 | 1 |
| **lpg2446** | 280 | 16 | 94 | - | - | 4 | 12 | 4 |
| **lpg2450** | 729 | 14 | 98 | - | - | 3 | 10 | 4 |
| **lpg2451** | 969 | 11 | 98 | - | - | 7 | 4 | 7 |
| **lpg2452** | 2767 | 21 | 99 | - | - | 8 | 12 | 9 |
| **lpg2453** | 450 | 12 | 97 | - | - | 6 | 6 | 6 |
| **lpg2454** | 448 | 11 | 97 | - | 1 | 6 | 5 | 6 |
| **lpg2529** | 1719 | 16 | 99 | - | - | 8 | 8 | 8 |
| **lpg2530** | 595 | 56 | 90 | 1 | - | 25 | 33 | 24 + 1 bp insertion |
| **lpg2534** | 432 | 21 | 95 | - | - | 2 | 18 | 3 |
| **lpg2535** | 949 | 14 | 98 | - | - | 6 | 8 | 6 |
