# Supplementary material for: Genome analysis of Legionella pneumophila ST23 from various countries reveals highly similar strains
Source: Life Sci Alliance. 2022 Mar 2;5(6):e202101117. doi: 10.26508/lsa.202101117 (PMC8899845; doi:10.26508/lsa.202101117)
Supplement: Supplementary file 5 [file LSA-2021-01117_TableS5.docx]

**Table S5.** cgMLST loci of difference found in 299C genome isolated in Verona

| **Target** | **Begin** | **End** | **Locus** | **GenBank protein_ID** | **Protein name** |
| --- | --- | --- | --- | --- | --- |
| lpg0004 | 4482 | 6902 | gyrB | [YP_094059.1](https://www.ncbi.nlm.nih.gov/protein/YP_094059.1) | DNA gyrase subunit B |
| lpg0006 | 8314 | 10206 | speA | [YP_094061.1](https://www.ncbi.nlm.nih.gov/protein/YP_094061.1) | arginine decarboxylase |
| lpg0007 | 10226 | 11083 | - | [YP_094062.1](https://www.ncbi.nlm.nih.gov/protein/YP_094062.1) | hydrolase |
| lpg0033 | 40298 | 41323 | - | [YP_094088.1](https://www.ncbi.nlm.nih.gov/protein/YP_094088.1) | hypothetical protein lpg0033 |
| lpg0084 | 93312 | 94835 | - | [YP_094138.1](https://www.ncbi.nlm.nih.gov/protein/YP_094138.1) | hypothetical protein lpg0084 |
| lpg0086 | 95581 | 97527 | - | [YP_094140.1](https://www.ncbi.nlm.nih.gov/protein/YP_094140.1) | hypothetical protein lpg0086 |
| lpg0100 | 114931 | 116001 | - | [YP_094154.1](https://www.ncbi.nlm.nih.gov/protein/YP_094154.1) | UDP-3-O |
| lpg0238 | 281131 | 282597 | gbsA | [YP_094292.1](https://www.ncbi.nlm.nih.gov/protein/YP_094292.1) | glycine betaine aldehyde dehydrogenase |
| lpg0625 | 658819 | 660174 | - | [YP_094661.1](https://www.ncbi.nlm.nih.gov/protein/YP_094661.1) | hypothetical protein lpg0625 |
| lpg0626 | 660200 | 662407 | - | [YP_094662.1](https://www.ncbi.nlm.nih.gov/protein/YP_094662.1) | DNA uptake/competence protein ComA |
| lpg0634 | 670559 | 671908 | - | [YP_094670.1](https://www.ncbi.nlm.nih.gov/protein/YP_094670.1) | hypothetical protein lpg0634 |
| lpg0693 | 748803 | 753083 | - | [YP_094729.1](https://www.ncbi.nlm.nih.gov/protein/YP_094729.1) | LigA, interaptin |
| lpg0891 | 964156 | 966471 | - | [YP_094925.1](https://www.ncbi.nlm.nih.gov/protein/YP_094925.1) | sensory box protein |
| lpg1369 | 1514897 | 1516768 | htpG | [YP_095398.1](https://www.ncbi.nlm.nih.gov/protein/YP_095398.1) | heat shock protein 90 |
| ≈13587bp | | | | | |
| lpg1373 | 1519616 | 1520191 | rnhB | [YP_095402.1](https://www.ncbi.nlm.nih.gov/protein/YP_095402.1) | ribonuclease HII |
| lpg1374 | 1520302 | 1521420 | rodA | [YP_095403.1](https://www.ncbi.nlm.nih.gov/protein/YP_095403.1) | rod shape determining protein RodA |
| lpg1375 | 1521417 | 1523297 | pbpA | [YP_095404.1](https://www.ncbi.nlm.nih.gov/protein/YP_095404.1) | penicillin binding protein 2 |
| lpg1376 | 1523437 | 1523907 | - | [YP_095405.1](https://www.ncbi.nlm.nih.gov/protein/YP_095405.1) | 23S rRNA (pseudouridine(1915)-N(3))-methyltransferase RlmH |
| lpg1378 | 1524359 | 1525801 | - | [YP_095407.1](https://www.ncbi.nlm.nih.gov/protein/YP_095407.1) | proton/peptide symporter, POT family protein |
| lpg1381 | 1529236 | 1530639 | - | [YP_095410.1](https://www.ncbi.nlm.nih.gov/protein/YP_095410.1) | aldehyde dehydrogenase |
| lpg1382 | 1530636 | 1531445 | - | [YP_095411.1](https://www.ncbi.nlm.nih.gov/protein/YP_095411.1) | short chain dehydrogenase |
| lpg1385 | 1532786 | 1533163 | - | [YP_095414.1](https://www.ncbi.nlm.nih.gov/protein/YP_095414.1) | hypothetical protein lpg1385 |
| ≈19197bp | | | | | |
| lpg1389 | 1535948 | 1537033 | mnmA | [YP_095418.1](https://www.ncbi.nlm.nih.gov/protein/YP_095418.1) | tRNA-specific 2-thiouridylase MnmA |
| lpg1394 | 1540022 | 1540969 | - | [YP_095423.1](https://www.ncbi.nlm.nih.gov/protein/YP_095423.1) | malonyl CoA-ACP transacylase |
| lpg1395 | 1540981 | 1541727 | fabG | [YP_095424.1](https://www.ncbi.nlm.nih.gov/protein/YP_095424.1) | 3-oxoacyl-ACP reductase |
| lpg1400 | 1544952 | 1545857 | holB | [YP_095429.1](https://www.ncbi.nlm.nih.gov/protein/YP_095429.1) | DNA polymerase III, delta prime subunit |
| lpg1401 | 1545984 | 1546325 | pilZ | [YP_095430.1](https://www.ncbi.nlm.nih.gov/protein/YP_095430.1) | type 4 fimbrial biogenesis protein PilZ |
| lpg1404 | 1548437 | 1549729 | - | [YP_095433.1](https://www.ncbi.nlm.nih.gov/protein/YP_095433.1) | major facilitator family transporter |
| lpg1406 | 1551307 | 1552482 | - | [YP_095435.1](https://www.ncbi.nlm.nih.gov/protein/YP_095435.1) | glycosyltransferase |
| lpg1407 | 1552519 | 1553904 | - | [YP_095436.1](https://www.ncbi.nlm.nih.gov/protein/YP_095436.1) | dolichol monophosphate mannose synthase |
| lpg1408 | 1553994 | 1555145 | licA | [YP_095437.1](https://www.ncbi.nlm.nih.gov/protein/YP_095437.1) | choline kinase |
| ≈10898bp | | | | | |
| lpg1536 | 1701541 | 1701963 | - | [YP_095565.1](https://www.ncbi.nlm.nih.gov/protein/YP_095565.1) | transmembrane protein |
| lpg1537 | 1701966 | 1702697 | - | [YP_095566.1](https://www.ncbi.nlm.nih.gov/protein/YP_095566.1) | transporter |
| lpg1538 | 1702965 | 1704275 | pcnB | [YP_095567.1](https://www.ncbi.nlm.nih.gov/protein/YP_095567.1) | poly(A) polymerase |
| lpg1540 | 1704752 | 1705174 | - | [YP_095569.1](https://www.ncbi.nlm.nih.gov/protein/YP_095569.1) | universal stress protein A |
| lpg1541 | 1705239 | 1706627 | engA | [YP_095570.1](https://www.ncbi.nlm.nih.gov/protein/YP_095570.1) | GTP-binding protein EngA |
| lpg1542 | 1706632 | 1707795 | - | [YP_095571.1](https://www.ncbi.nlm.nih.gov/protein/YP_095571.1) | PQQ WD-40-like repeat-containing protein |
| lpg1547 | 1711279 | 1712439 | - | [YP_095576.1](https://www.ncbi.nlm.nih.gov/protein/YP_095576.1) | radical SAM protein |
|  |  |  |  |  |  |
| lpg1576 | 1741547 | 1742578 | ruvB | [YP_095605.1](https://www.ncbi.nlm.nih.gov/protein/YP_095605.1) | Holliday junction DNA helicase RuvB |
| lpg1652 | 1826195 | 1827199 | - | [YP_095679.1](https://www.ncbi.nlm.nih.gov/protein/YP_095679.1) | myo-inositol-2-dehydrogenase |
| lpg1655 | 1830346 | 1832019 | lasB | [YP_095682.1](https://www.ncbi.nlm.nih.gov/protein/YP_095682.1) | class 4 metalloprotease |
| lpg1665 | 1843217 | 1848982 | - | [YP_095692.1](https://www.ncbi.nlm.nih.gov/protein/YP_095692.1) | hypothetical protein lpg1665 |
| lpg1666 | 1849247 | 1850650 | - | [YP_095693.1](https://www.ncbi.nlm.nih.gov/protein/YP_095693.1) | hypothetical protein lpg1666 |
| lpg1673 | 1860352 | 1861671 | - | [YP_095700.1](https://www.ncbi.nlm.nih.gov/protein/YP_095700.1) | phosphoribosylamine-glycine ligase |
| lpg1680 | 1870228 | 1871607 | dsbD | [YP_095707.1](https://www.ncbi.nlm.nih.gov/protein/YP_095707.1) | thiol:disulfide interchange protein DsbD |
| lpg1690 | 1881178 | 1883853 | acnA | [YP_095717.1](https://www.ncbi.nlm.nih.gov/protein/YP_095717.1) | aconitate hydratase |
| lpg1696 | 1888202 | 1891366 | putA | [YP_095723.1](https://www.ncbi.nlm.nih.gov/protein/YP_095723.1) | bifunctional proline dehydrogenase/pyrroline-5-carboxylate dehydrogenase |
|  | | | | | |
| lpg1707 | 1902841 | 1904331 | astD | [YP_095734.1](https://www.ncbi.nlm.nih.gov/protein/YP_095734.1) | succinylglutamic semialdehyde dehydrogenase |
| lpg1708 | 1904337 | 1905683 | astB | [YP_095735.1](https://www.ncbi.nlm.nih.gov/protein/YP_095735.1) | succinylarginine dihydrolase |
| lpg1710 | 1906498 | 1906863 | - | [YP_095737.1](https://www.ncbi.nlm.nih.gov/protein/YP_095737.1) | hypothetical protein lpg1710 |
| lpg1713 | 1908528 | 1909406 | tsf | [YP_095740.2](https://www.ncbi.nlm.nih.gov/protein/YP_095740.2) | elongation factor Ts |
| lpg1714 | 1909472 | 1910236 | rpsB | [YP_095741.2](https://www.ncbi.nlm.nih.gov/protein/YP_095741.2) | 30S ribosomal protein S2 |
|  |  |  |  |  |  |
| lpg1785 | 1992531 | 1994609 | flhA | [YP_095811.1](https://www.ncbi.nlm.nih.gov/protein/YP_095811.1) | flagellar biosynthesis protein FlhA |
| lpg1787 | 1995754 | 1996524 | fliR | [YP_095813.1](https://www.ncbi.nlm.nih.gov/protein/YP_095813.1) | flagellar biosynthetic protein FliR |
| lpg1789 | 1996806 | 1997555 | fliP | [YP_095815.1](https://www.ncbi.nlm.nih.gov/protein/YP_095815.1) | flagellar biosynthesis protein FliP |
| lpg1792 | 1998302 | 1999369 | fliM | [YP_095818.1](https://www.ncbi.nlm.nih.gov/protein/YP_095818.1) | flagellar motor switch protein FliM |
|  |  |  |  |  |  |
| lpg1799 | 2007376 | 2009958 | alaS | [YP_095825.1](https://www.ncbi.nlm.nih.gov/protein/YP_095825.1) | alanyl-tRNA synthetase |
| lpg1801 | 2010421 | 2011467 | recA | [YP_095827.1](https://www.ncbi.nlm.nih.gov/protein/YP_095827.1) | recombinase A |
| lpg1803 | 2011770 | 2012705 | - | [YP_095829.1](https://www.ncbi.nlm.nih.gov/protein/YP_095829.1) | hypothetical protein lpg1803 |
| lpg1805 | 2014053 | 2016650 | mutS | [YP_095831.1](https://www.ncbi.nlm.nih.gov/protein/YP_095831.1) | DNA mismatch repair protein MutS |
| lpg1806 | 2016663 | 2018351 | - | [YP_095832.1](https://www.ncbi.nlm.nih.gov/protein/YP_095832.1) | hypothetical protein lpg1806 |
| lpg1811 | 2024508 | 2027087 | lysC | [YP_095837.1](https://www.ncbi.nlm.nih.gov/protein/YP_095837.1) | bifunctional aspartate kinase/diaminopimelate decarboxylase |
| ≈21165bp | | | | | |
| lpg1823 | 2041557 | 2042243 | - | [YP_095849.1](https://www.ncbi.nlm.nih.gov/protein/YP_095849.1) | hypothetical protein lpg1823 |
| lpg1824 | 2042569 | 2043738 | - | [YP_095850.1](https://www.ncbi.nlm.nih.gov/protein/YP_095850.1) | acyl CoA dehydrogenase |
| lpg1825 | 2043750 | 2044934 | atoB | [YP_095851.1](https://www.ncbi.nlm.nih.gov/protein/YP_095851.1) | acyl CoA C-acetyltransferase |
| lpg1827 | 2045396 | 2047003 | - | [YP_095853.1](https://www.ncbi.nlm.nih.gov/protein/YP_095853.1) | propionyl CoA carboxylase subunit beta |
| lpg1829 | 2047741 | 2049774 | - | [YP_095855.1](https://www.ncbi.nlm.nih.gov/protein/YP_095855.1) | acyl CoA carboxylase subunit alpha |
| lpg1830 | 2049785 | 2050693 | mvaB | [YP_095856.1](https://www.ncbi.nlm.nih.gov/protein/YP_095856.1) | hydroxymethylglutaryl-CoA lyase |
| lpg1831 | 2050790 | 2052790 | - | [YP_095857.1](https://www.ncbi.nlm.nih.gov/protein/YP_095857.1) | acetoacetyl-CoA synthetase |
| lpg1836 | 2056349 | 2057767 | - | [YP_095862.1](https://www.ncbi.nlm.nih.gov/protein/YP_095862.1) | coiled coil domain-containing protein |
| lpg1838 | 2058990 | 2059520 | - | [YP_095864.1](https://www.ncbi.nlm.nih.gov/protein/YP_095864.1) | D,D-heptose 1,7-bisphosphate phosphatase |
| lpg1841 | 2062722 | 2063507 | com1 | [YP_095867.1](https://www.ncbi.nlm.nih.gov/protein/YP_095867.1) | hypothetical protein lpg1841 |
|  |  |  |  |  |  |
| lpg1892 | 2110192 | 2110575 | - | [YP_095909.1](https://www.ncbi.nlm.nih.gov/protein/YP_095909.1) | hypothetical protein lpg1892 |
| lpg1893 | 2110585 | 2111865 | - | [YP_095910.1](https://www.ncbi.nlm.nih.gov/protein/YP_095910.1) | major facilitator family transporter |
| lpg1894 | 2112309 | 2113622 | - | [YP_095911.1](https://www.ncbi.nlm.nih.gov/protein/YP_095911.1) | chloride channel protein (voltage gated) |
| lpg2508 | 2827446 | 2829869 | - | [YP_096515.1](https://www.ncbi.nlm.nih.gov/protein/YP_096515.1) | hypothetical protein lpg2508 |
| lpg2512 | 2837380 | 2840532 | - | [YP_096519.1](https://www.ncbi.nlm.nih.gov/protein/YP_096519.1) | RND multidrug efflux transporter MexF |
| lpg2514 | 2841752 | 2843314 | - | [YP_096521.1](https://www.ncbi.nlm.nih.gov/protein/YP_096521.1) | outer membrane efflux protein |
| lpg2518 | 2845967 | 2846308 | - | [YP_096525.1](https://www.ncbi.nlm.nih.gov/protein/YP_096525.1) | hypothetical protein lpg2518 |
| lpg2526 | 2854590 | 2855957 | - | [YP_096533.1](https://www.ncbi.nlm.nih.gov/protein/YP_096533.1) | hypothetical protein lpg2526 |
| lpg2530 | 2862296 | 2863339 | aroF | [YP_096537.1](https://www.ncbi.nlm.nih.gov/protein/YP_096537.1) | 3-deoxy-7-phosphoheptulonate synthase |
| lpg2534 | 2866611 | 2867042 | - | [YP_096541.1](https://www.ncbi.nlm.nih.gov/protein/YP_096541.1) | hypothetical protein lpg2534 |
| lpg2535 | 2867089 | 2867496 | - | [YP_096542.1](https://www.ncbi.nlm.nih.gov/protein/YP_096542.1) | hypothetical protein lpg2535 |
| lpg2544 | 2875791 | 2877164 | mltA | [YP_096551.1](https://www.ncbi.nlm.nih.gov/protein/YP_096551.1) | membrane-bound lytic murein transglycosylase A |
| lpg2553 | 2885982 | 2887349 | uhpC | [YP_096560.1](https://www.ncbi.nlm.nih.gov/protein/YP_096560.1) | hexosephosphate transport |
| lpg2577 | 2905863 | 2906621 | - | [YP_096582.1](https://www.ncbi.nlm.nih.gov/protein/YP_096582.1) | hypothetical protein lpg2577 |
| lpg2579 | 2907214 | 2907627 | - | [YP_096584.1](https://www.ncbi.nlm.nih.gov/protein/YP_096584.1) | hypothetical protein lpg2579 |
| lpg2581 | 2908957 | 2911227 | - | [YP_096586.1](https://www.ncbi.nlm.nih.gov/protein/YP_096586.1) | 2-oxoisovalerate dehydrogenase E1 |
| lpg2582 | 2911432 | 2912598 | - | [YP_096587.1](https://www.ncbi.nlm.nih.gov/protein/YP_096587.1) | hypothetical protein lpg2582 |
| ≈64862bp | | | | | |
| lpg2589 | 2921870 | 2923663 | dacB | [YP_096594.1](https://www.ncbi.nlm.nih.gov/protein/YP_096594.1) | D-alanyl-D-alanine carboxypeptidase |
| lpg2590 | 2923755 | 2924546 | spoOJ | [YP_096595.1](https://www.ncbi.nlm.nih.gov/protein/YP_096595.1) | chromosome partitioning protein ParB |
| lpg2594 | 2927359 | 2928303 | fmt | [YP_096599.1](https://www.ncbi.nlm.nih.gov/protein/YP_096599.1) | methionyl tRNA formyltransferase |
| lpg2596 | 2928906 | 2929943 | - | [YP_096601.1](https://www.ncbi.nlm.nih.gov/protein/YP_096601.1) | signal peptide protein |
| lpg2597 | 2929940 | 2931025 | - | [YP_096602.1](https://www.ncbi.nlm.nih.gov/protein/YP_096602.1) | DNA processing protein DprA |
| lpg2598 | 2931036 | 2931452 | - | [YP_096603.1](https://www.ncbi.nlm.nih.gov/protein/YP_096603.1) | hypothetical protein lpg2598 |
| lpg2599 | 2931531 | 2933810 | topA | [YP_096604.1](https://www.ncbi.nlm.nih.gov/protein/YP_096604.1) | DNA topoisomerase I |
| lpg2602 | 2936592 | 2937014 | - | [YP_096607.1](https://www.ncbi.nlm.nih.gov/protein/YP_096607.1) | hypothetical protein lpg2602 |
| lpg2604 | 2938631 | 2939434 | - | [YP_096609.1](https://www.ncbi.nlm.nih.gov/protein/YP_096609.1) | hypothetical protein lpg2604 |
| lpg2606 | 2940021 | 2940887 | - | [YP_096611.1](https://www.ncbi.nlm.nih.gov/protein/YP_096611.1) | glutamine amidotransferase |
| lpg2607 | 2941026 | 2943062 | pepO | [YP_096612.1](https://www.ncbi.nlm.nih.gov/protein/YP_096612.1) | metallopeptidase PepO |
| lpg2608 | 2943206 | 2944120 | lpxC | [YP_096613.1](https://www.ncbi.nlm.nih.gov/protein/YP_096613.1) | UDP-3-O |
| lpg2609 | 2944368 | 2945564 | ftsZ | [YP_096614.1](https://www.ncbi.nlm.nih.gov/protein/YP_096614.1) | cell division protein FtsZ |
| lpg2610 | 2945759 | 2947021 | ftsA | [YP_096615.1](https://www.ncbi.nlm.nih.gov/protein/YP_096615.1) | ATP-binding cell division protein FtsA |
| lpg2615 | 2951164 | 2952348 | ftsW | [YP_096620.1](https://www.ncbi.nlm.nih.gov/protein/YP_096620.1) | cell division protein FtsW |
| lpg2616 | 2952345 | 2953688 | murD | [YP_096621.1](https://www.ncbi.nlm.nih.gov/protein/YP_096621.1) | UDP-N-acetylmuramoyl-L-alanyl-D-glutamate synthetase |
| lpg2617 | 2953702 | 2954820 | mraY | [YP_096622.1](https://www.ncbi.nlm.nih.gov/protein/YP_096622.1) | phospho-N-acetylmuramoyl-pentapeptide-transferase |
| lpg2618 | 2954902 | 2956287 | murF | [YP_096623.1](https://www.ncbi.nlm.nih.gov/protein/YP_096623.1) | UDP-N-acetylmuramoyl-tripeptide--D-alanyl-D-alanine ligase |
| lpg2620 | 2957264 | 2960758 | - | [YP_096625.1](https://www.ncbi.nlm.nih.gov/protein/YP_096625.1) | chromosome segregation protein SMC |
| lpg2621 | 2960933 | 2961613 | - | [YP_096626.1](https://www.ncbi.nlm.nih.gov/protein/YP_096626.1) | acid phosphatase, class B |
| lpg2622 | 2961715 | 2962776 | - | [YP_096627.1](https://www.ncbi.nlm.nih.gov/protein/YP_096627.1) | hypothetical protein lpg2622 |
| lpg2623 | 2963086 | 2963898 | - | [YP_096628.1](https://www.ncbi.nlm.nih.gov/protein/YP_096628.1) | transmembrane protein |
| lpg2624 | 2963973 | 2964455 | greA | [YP_096629.1](https://www.ncbi.nlm.nih.gov/protein/YP_096629.1) | transcription elongation factor GreA |
| lpg2625 | 2964464 | 2967667 | carB | [YP_096630.1](https://www.ncbi.nlm.nih.gov/protein/YP_096630.1) | carbamoyl phosphate synthase, large subunit |
| lpg2626 | 2967794 | 2968066 | - | [YP_096631.1](https://www.ncbi.nlm.nih.gov/protein/YP_096631.1) | hypothetical protein lpg2626 |
| lpg2627 | 2968179 | 2969360 | - | [YP_096632.1](https://www.ncbi.nlm.nih.gov/protein/YP_096632.1) | hypothetical protein lpg2627 |
| lpg2631 | 2972463 | 2973947 | pepA | [YP_096636.1](https://www.ncbi.nlm.nih.gov/protein/YP_096636.1) | aminopeptidase |
| lpg2633 | 2974501 | 2974818 | - | [YP_096638.1](https://www.ncbi.nlm.nih.gov/protein/YP_096638.1) | hypothetical protein lpg2633 |
| lpg2635 | 2976210 | 2977781 | mviN | [YP_096640.1](https://www.ncbi.nlm.nih.gov/protein/YP_096640.1) | virulence factor MviN |
| lpg2639 | 2981818 | 2985420 | enhC | [YP_096644.1](https://www.ncbi.nlm.nih.gov/protein/YP_096644.1) | enhanced entry protein EnhC |
| lpg2641 | 2986010 | 2986732 | enhA | [YP_096646.1](https://www.ncbi.nlm.nih.gov/protein/YP_096646.1) | enhanced entry protein EnhA |
|  |  |  |  |  |  |
| lpg2645 | 2991885 | 2993741 | uvrC | [YP_096650.1](https://www.ncbi.nlm.nih.gov/protein/YP_096650.1) | excinuclease ABC subunit C |
| lpg2651 | 2998381 | 2998692 | rplU | [YP_096656.1](https://www.ncbi.nlm.nih.gov/protein/YP_096656.1) | 50S ribosomal protein L21 |
| ≈31667bp | | | | | |
| lpg2655 | 3001753 | 3002868 | - | [YP_096660.1](https://www.ncbi.nlm.nih.gov/protein/YP_096660.1) | sensory box protein |
| lpg2657 | 3004193 | 3006448 | feoB | [YP_096662.1](https://www.ncbi.nlm.nih.gov/protein/YP_096662.1) | ferrous iron transport protein B |
| lpg2658 | 3006445 | 3006672 | feoA | [YP_096663.1](https://www.ncbi.nlm.nih.gov/protein/YP_096663.1) | ferrous iron transporter A |
| lpg2659 | 3006761 | 3007852 | - | [YP_096664.1](https://www.ncbi.nlm.nih.gov/protein/YP_096664.1) | ATPase N2B (nucleotide (GTP) binding protein) |
| lpg2662 | 3009539 | 3010297 | panC | [YP_096667.1](https://www.ncbi.nlm.nih.gov/protein/YP_096667.1) | pantoate-beta-alanine ligase |
| lpg2666 | 3013221 | 3014102 | - | [YP_096671.1](https://www.ncbi.nlm.nih.gov/protein/YP_096671.1) | hydrolase |
| lpg2667 | 3014236 | 3015114 | rpoH | [YP_096672.1](https://www.ncbi.nlm.nih.gov/protein/YP_096672.1) | RNA polymerase factor sigma-32 |
| lpg2668 | 3015387 | 3016316 | ftsX | [YP_096673.1](https://www.ncbi.nlm.nih.gov/protein/YP_096673.1) | cell division ATP transporter FtsX |
| lpg2671 | 3018077 | 3019402 | - | [YP_096676.1](https://www.ncbi.nlm.nih.gov/protein/YP_096676.1) | zinc protease |
| lpg2672 | 3019399 | 3020703 | - | [YP_096677.1](https://www.ncbi.nlm.nih.gov/protein/YP_096677.1) | zinc protease |
| lpg2673 | 3020700 | 3021245 | - | [YP_096678.1](https://www.ncbi.nlm.nih.gov/protein/YP_096678.1) | N-6 adenine-specific DNA methylase |
| lpg2676 | 3022673 | 3023836 | dotB | [YP_096681.1](https://www.ncbi.nlm.nih.gov/protein/YP_096681.1) | ATPase |
| lpg2678 | 3025688 | 3026485 | - | [YP_096683.1](https://www.ncbi.nlm.nih.gov/protein/YP_096683.1) | hypothetical protein lpg2678 |
| lpg2680 | 3027690 | 3028718 | murE3 | [YP_096685.1](https://www.ncbi.nlm.nih.gov/protein/YP_096685.1) | UDP-N-acetylmuramyl tripeptide synthase |
| lpg2684 | 3032560 | 3033420 | - | [YP_096689.1](https://www.ncbi.nlm.nih.gov/protein/YP_096689.1) | hypothetical protein lpg2684 |
| ≈45659bp | | | | | |
| lpg2817 | 3179864 | 3180727 | yrfI | [YP_096812.1](https://www.ncbi.nlm.nih.gov/protein/YP_096812.1) | heat shock protein 33, redox regulated chaperonin |
| lpg2818 | 3180741 | 3181238 | - | [YP_096813.1](https://www.ncbi.nlm.nih.gov/protein/YP_096813.1) | hypothetical protein lpg2818 |
| lpg2819 | 3181506 | 3182465 | - | [YP_096814.1](https://www.ncbi.nlm.nih.gov/protein/YP_096814.1) | tyrosine phosphatase II superfamily protein |
| lpg2822 | 3184685 | 3186511 | bipA | [YP_096817.1](https://www.ncbi.nlm.nih.gov/protein/YP_096817.1) | virulence regulator BipA |
| lpg2823 | 3186518 | 3187405 | ppnK | [YP_096818.1](https://www.ncbi.nlm.nih.gov/protein/YP_096818.1) | inorganic polyphosphate/ATP-NAD kinase |
| lpg2824 | 3187409 | 3189076 | recN | [YP_096819.1](https://www.ncbi.nlm.nih.gov/protein/YP_096819.1) | DNA repair protein RecN |
| lpg2827 | 3191867 | 3192844 | - | [YP_096822.1](https://www.ncbi.nlm.nih.gov/protein/YP_096822.1) | hypothetical protein lpg2827 |
| lpg2832 | 3204025 | 3205665 | - | [YP_096827.1](https://www.ncbi.nlm.nih.gov/protein/YP_096827.1) | hypothetical protein lpg2832 |
| lpg2833 | 3205662 | 3206042 | yciA | [YP_096828.1](https://www.ncbi.nlm.nih.gov/protein/YP_096828.1) | acyl-CoA thioesterase |
| lpg2834 | 3206045 | 3208414 | - | [YP_096829.1](https://www.ncbi.nlm.nih.gov/protein/YP_096829.1) | transcription accessory protein |
| lpg2836 | 3209200 | 3211014 | glmS | [YP_096831.1](https://www.ncbi.nlm.nih.gov/protein/YP_096831.1) | glucosamine--fructose-6-phosphate aminotransferase |
| lpg2837 | 3211378 | 3212679 | - | [YP_096832.1](https://www.ncbi.nlm.nih.gov/protein/YP_096832.1) | phospholipase/lecithinase/hemolysin, lysophospholipase A, glycerophospholipid-cholesterol acyltransf... |
| lpg2838 | 3212932 | 3213696 | - | [YP_096833.1](https://www.ncbi.nlm.nih.gov/protein/YP_096833.1) | rhodanese domain-containing protein |
| lpg2840 | 3214236 | 3214754 | - | [YP_096835.1](https://www.ncbi.nlm.nih.gov/protein/YP_096835.1) | peroxiredoxin, AhpC/TSA family protein |
| lpg2841 | 3214880 | 3216079 | - | YP_096836.1 | major facilitator transporter |
| lpg2842 | 3216234 | 3217640 | - | YP_096837.1 | PhoH protein (phosphate starvation inducible protein) |
| lpg2843 | 3217663 | 3218676 | - | [YP_096838.1](https://www.ncbi.nlm.nih.gov/protein/YP_096838.1) | inosine 5\'-monophosphate dehydrogenase |
| lpg2847 | 3221661 | 3222623 | - | [YP_096842.1](https://www.ncbi.nlm.nih.gov/protein/YP_096842.1) | hypothetical protein lpg2847 |
| lpg2848 | 3222761 | 3223774 | - | [YP_096843.1](https://www.ncbi.nlm.nih.gov/protein/YP_096843.1) | ribonuclease T2 family protein |
| lpg2850 | 3225308 | 3225523 | - | [YP_096845.1](https://www.ncbi.nlm.nih.gov/protein/YP_096845.1) | cold shock transcriptional regulator CspA |
|  |  |  |  |  |  |
| lpg2976 | 3366946 | 3368475 | - | [YP_096968.1](https://www.ncbi.nlm.nih.gov/protein/YP_096968.1) | hypothetical protein lpg2976 |
| ≈18643bp | | | | | |
| lpg2983 | 3376631 | 3377497 | atpG | [YP_096975.1](https://www.ncbi.nlm.nih.gov/protein/YP_096975.1) | ATP synthase F0F1 subunit gamma |
| lpg2984 | 3377587 | 3379140 | - | [YP_096976.1](https://www.ncbi.nlm.nih.gov/protein/YP_096976.1) | ATP synthase F0F1 subunit alpha |
| lpg2985 | 3379158 | 3379715 | atpH | [YP_096977.1](https://www.ncbi.nlm.nih.gov/protein/YP_096977.1) | ATP synthase F0F1 subunit delta |
| lpg2988 | 3380540 | 3381418 | atpB | [YP_096980.1](https://www.ncbi.nlm.nih.gov/protein/YP_096980.1) | ATP synthase F0F1 subunit A |
| lpg2992 | 3382822 | 3383394 | - | [YP_096984.1](https://www.ncbi.nlm.nih.gov/protein/YP_096984.1) | hypothetical protein lpg2992 |
| lpg2993 | 3383398 | 3383997 | gmhA | [YP_096985.1](https://www.ncbi.nlm.nih.gov/protein/YP_096985.1) | hypothetical protein lpg2993 |
| lpg2994 | 3384024 | 3384380 | - | [YP_096986.1](https://www.ncbi.nlm.nih.gov/protein/YP_096986.1) | hypothetical protein lpg2994 |
| lpg2995 | 3384402 | 3386213 | - | [YP_096987.1](https://www.ncbi.nlm.nih.gov/protein/YP_096987.1) | lipoprotein |
| lpg2997 | 3387135 | 3388301 | - | [YP_096989.1](https://www.ncbi.nlm.nih.gov/protein/YP_096989.1) | alkane-1 monooxygenase |
| lpg2998 | 3388349 | 3390526 | - | [YP_096990.1](https://www.ncbi.nlm.nih.gov/protein/YP_096990.1) | sulfate transporter |
| lpg2999 | 3391003 | 3391803 | legP | [YP_096991.1](https://www.ncbi.nlm.nih.gov/protein/YP_096991.1) | hypothetical protein lpg2999 |
| lpg3001 | 3393934 | 3395274 | trmE | [YP_096993.1](https://www.ncbi.nlm.nih.gov/protein/YP_096993.1) | tRNA modification GTPase TrmE |
