# Supplementary material for: Genome analysis of Legionella pneumophila ST23 from various countries reveals highly similar strains
Source: Life Sci Alliance. 2022 Mar 2;5(6):e202101117. doi: 10.26508/lsa.202101117 (PMC8899845; doi:10.26508/lsa.202101117)
Supplement: Supplementary file 6 [file LSA-2021-01117_TableS6.docx]

**Table S6.** cgMLST loci of difference found in 1C genome isolated in Rome

| **Target** | **Begin** | **End** | **Locus** | **GenBank protein_ID** | **Protein name** |
| --- | --- | --- | --- | --- | --- |
| lpg0370 | 409989 | 410423 | + | [YP_094414.1](https://www.ncbi.nlm.nih.gov/protein/YP_094414.1) | oligoketide cyclase/lipid transporter protein |
| lpg0400 | 438585 | 439961 | ffh | [YP_094444.1](https://www.ncbi.nlm.nih.gov/protein/YP_094444.1) | signal recognition particle protein Ffh |
| lpg0401 | 440213 | 440887 | - | [YP_094445.1](https://www.ncbi.nlm.nih.gov/protein/YP_094445.1) | hypothetical protein lpg0401 |
| lpg0404 | 445085 | 446488 | - | [YP_094448.1](https://www.ncbi.nlm.nih.gov/protein/YP_094448.1) | amino acid antiporter |
| lpg0513 | 553352 | 554632 | serS | [YP_094557.1](https://www.ncbi.nlm.nih.gov/protein/YP_094557.1) | seryl-tRNA synthetase |
| lpg0517 | 558434 | 559264 | ytbE | [YP_094561.1](https://www.ncbi.nlm.nih.gov/protein/YP_094561.1) | aldo/keto reductase |
| lpg0518 | 559574 | 560422 | - | [YP_094562.1](https://www.ncbi.nlm.nih.gov/protein/YP_094562.1) | hypothetical protein lpg0518 |
| lpg0841 | 919557 | 920354 | - | [YP_094876.1](https://www.ncbi.nlm.nih.gov/protein/YP_094876.1) | ABC transporter ATP-binding protein |
| lpg1274 | 1402531 | 1402986 | - | [YP_095304.1](https://www.ncbi.nlm.nih.gov/protein/YP_095304.1) | hypothetical protein lpg1274 |
| ≈8000bp | | | | | |
| lpg1279 | 1407804 | 1408175 | - | [YP_095309.1](https://www.ncbi.nlm.nih.gov/protein/YP_095309.1) | hypothetical protein lpg1279 |
| lpg1280 | 1408223 | 1409947 | sfcA | [YP_095310.1](https://www.ncbi.nlm.nih.gov/protein/YP_095310.1) | malate dehydrogenase |
| lpg1281 | 1410027 | 1410410 | - | [YP_095311.1](https://www.ncbi.nlm.nih.gov/protein/YP_095311.1) | hypothetical protein lpg1281 |
| lpg1282 | 1410413 | 1411168 | surE | [YP_095312.1](https://www.ncbi.nlm.nih.gov/protein/YP_095312.1) | 5\'-nucleotidase SurE |
| lpg1283 | 1411187 | 1411930 | - | [YP_095313.1](https://www.ncbi.nlm.nih.gov/protein/YP_095313.1) | lipoprotein NlpD |
| lpg1284 | 1411962 | 1413041 | rpoS | [YP_095314.1](https://www.ncbi.nlm.nih.gov/protein/YP_095314.1) | stationary phase specific sigma factor RpoS |
| lpg1285 | 1413144 | 1414394 | - | [YP_095315.1](https://www.ncbi.nlm.nih.gov/protein/YP_095315.1) | homogentisate 1,2-dioxygenase |
| lpg1286 | 1414540 | 1415283 | - | [YP_095316.1](https://www.ncbi.nlm.nih.gov/protein/YP_095316.1) | hypothetical protein lpg1286 |
| lpg1287 | 1415283 | 1415807 | ruvC | [YP_095317.1](https://www.ncbi.nlm.nih.gov/protein/YP_095317.1) | Holliday junction resolvase |
|  |  |  |  |  |  |
| lpg1778 | 1986556 | 1987563 | prfB | [YP_095804.1](https://www.ncbi.nlm.nih.gov/protein/YP_095804.1) | peptide chain release factor 2 |
| lpg1779 | 1987723 | 1988124 | - | [YP_095805.1](https://www.ncbi.nlm.nih.gov/protein/YP_095805.1) | hypothetical protein lpg1779 |
| lpg1780 | 1988121 | 1988942 | motB | [YP_095806.1](https://www.ncbi.nlm.nih.gov/protein/YP_095806.1) | flagellar motor protein MotD |
| lpg1782 | 1989682 | 1990398 | fliA | [YP_095808.2](https://www.ncbi.nlm.nih.gov/protein/YP_095808.2) | flagellar biosynthesis sigma factor |
| lpg1993 | 2233519 | 2234397 | - | [YP_096009.1](https://www.ncbi.nlm.nih.gov/protein/YP_096009.1) | polysaccharide deacetylase |
| lpg2193 | 2475808 | 2477361 | - | [YP_096205.1](https://www.ncbi.nlm.nih.gov/protein/YP_096205.1) | sulfate transporter |
| lpg2194 | 2477365 | 2477991 | - | [YP_096206.1](https://www.ncbi.nlm.nih.gov/protein/YP_096206.1) | (beta)-carbonic anhydrase |
| lpg2196 | 2478431 | 2479402 | - | [YP_096208.1](https://www.ncbi.nlm.nih.gov/protein/YP_096208.1) | ornithine cyclodeaminase |
| ≈19477bp | | | | | |
| lpg2345 | 2648364 | 2650133 | deaD | [YP_096354.1](https://www.ncbi.nlm.nih.gov/protein/YP_096354.1) | ATP-dependent RNA helicase |
| lpg2347 | 2651415 | 2653439 | fadH | [YP_096356.1](https://www.ncbi.nlm.nih.gov/protein/YP_096356.1) | 2,4-dienoyl-CoA reductase |
| lpg2348 | 2653744 | 2654232 | sodC | [YP_096357.1](https://www.ncbi.nlm.nih.gov/protein/YP_096357.1) | superoxide dismutase |
| lpg2349 | 2654276 | 2654791 | - | [YP_096358.2](https://www.ncbi.nlm.nih.gov/protein/YP_096358.2) | alkylhydroperoxidase |
| lpg2352 | 2656503 | 2657495 | mdh | [YP_096361.1](https://www.ncbi.nlm.nih.gov/protein/YP_096361.1) | malate dehydrogenase |
| lpg2354 | 2658127 | 2659254 | - | [YP_096363.1](https://www.ncbi.nlm.nih.gov/protein/YP_096363.1) | (oxygen-independent) coproporphyrinogen III oxidase |
| lpg2356 | 2661049 | 2661882 | - | [YP_096365.1](https://www.ncbi.nlm.nih.gov/protein/YP_096365.1) | hypothetical protein lpg2356 |
| lpg2357 | 2662056 | 2663057 | gcp | [YP_096366.1](https://www.ncbi.nlm.nih.gov/protein/YP_096366.1) | DNA-binding/iron metalloprotein/AP endonuclease |
| lpg2361 | 2665970 | 2667841 | rpoD | [YP_096370.1](https://www.ncbi.nlm.nih.gov/protein/YP_096370.1) | RNA polymerase sigma 70 factor (RpoD) |
| lpg2372 | 2677194 | 2678462 | - | [YP_096380.1](https://www.ncbi.nlm.nih.gov/protein/YP_096380.1) | hypothetical protein lpg2372 |
| lpg2373 | 2679203 | 2679874 | - | [YP_096381.1](https://www.ncbi.nlm.nih.gov/protein/YP_096381.1) | hypothetical protein lpg2373 |
| ≈13819 bp | | | | | |
| lpg2481 | 2794324 | 2795226 | - | [YP_096488.1](https://www.ncbi.nlm.nih.gov/protein/YP_096488.1) | hypothetical protein lpg2481 |
| lpg2485 | 2798044 | 2799759 | - | [YP_096492.1](https://www.ncbi.nlm.nih.gov/protein/YP_096492.1) | hypothetical protein lpg2485 |
| lpg2486 | 2800077 | 2801465 | - | [YP_096493.1](https://www.ncbi.nlm.nih.gov/protein/YP_096493.1) | phosphomannomutase |
| lpg2487 | 2801466 | 2801936 | dut | [YP_096494.1](https://www.ncbi.nlm.nih.gov/protein/YP_096494.1) | deoxyuridine 5\'-triphosphate nucleotidohydrolase |
| lpg2488 | 2801951 | 2803339 | - | [YP_096495.1](https://www.ncbi.nlm.nih.gov/protein/YP_096495.1) | bifunctional phosphopantothenoylcysteine decarboxylase/phosphopantothenate synthase |
| lpg2490 | 2804259 | 2808143 | lepB | [YP_096497.1](https://www.ncbi.nlm.nih.gov/protein/YP_096497.1) | hypothetical protein lpg2490 |
| lpg2547 | 2880193 | 2880528 | csaA | [YP_096554.1](https://www.ncbi.nlm.nih.gov/protein/YP_096554.1) | chaperonin CsaA |
| lpg2549 | 2881518 | 2882288 | - | [YP_096556.1](https://www.ncbi.nlm.nih.gov/protein/YP_096556.1) | AraC family transcriptional regulator |
