# Supplementary material for: Genome analysis of Legionella pneumophila ST23 from various countries reveals highly similar strains
Source: Life Sci Alliance. 2022 Mar 2;5(6):e202101117. doi: 10.26508/lsa.202101117 (PMC8899845; doi:10.26508/lsa.202101117)
Supplement: Supplementary file 7 [file LSA-2021-01117_TableS7.docx]

**Table S7.** cgMLST loci of difference found in 325C genome isolated in Bolzano

| **Target** | **Begin** | **End** | **Locus** | **GenBank protein_ID** | **Protein name** |
| --- | --- | --- | --- | --- | --- |
| lpg0590 | 623078 | 624589 | comM | YP_094626.1 | competence related protein ComM |
| lpg0591 | 624654 | 624914 | - | [YP_094627.1](https://www.ncbi.nlm.nih.gov/protein/YP_094627.1) | hypothetical protein lpg0591 |
| lpg0592 | 625023 | 625397 | glnK | [YP_094628.1](https://www.ncbi.nlm.nih.gov/protein/YP_094628.1) | nitrogen regulatory P-II transcription regulator |
| lpg0595 | 626434 | 627249 | - | [YP_094631.1](https://www.ncbi.nlm.nih.gov/protein/YP_094631.1) | 4-amino-4-deoxychorismate lyase |
| lpg0596 | 627276 | 627971 | - | [YP_094632.1](https://www.ncbi.nlm.nih.gov/protein/YP_094632.1) | hypothetical protein lpg0596 |
| lpg1148 | 1264367 | 1265878 | lupA | [YP_095181.1](https://www.ncbi.nlm.nih.gov/protein/YP_095181.1) | ubiquitin-specific protease A |
| lpg1203 | 1331410 | 1332546 | cydB | [YP_095234.1](https://www.ncbi.nlm.nih.gov/protein/YP_095234.1) | cytochrome D ubiquinol oxidase subunit II |
| lpg1220 | 1345768 | 1346514 | flgF | [YP_095251.1](https://www.ncbi.nlm.nih.gov/protein/YP_095251.1) | flagellar basal body rod protein FlgF |
| lpg1276 | 1403689 | 1405320 | - | [YP_095306.1](https://www.ncbi.nlm.nih.gov/protein/YP_095306.1) | electron transferring flavoprotein dehydrogenase |
| lpg1655 | 1830346 | 1832019 | lasB | [YP_095682.1](https://www.ncbi.nlm.nih.gov/protein/YP_095682.1) | class 4 metalloprotease |
| lpg1665 | 1843217 | 1848982 | - | [YP_095692.1](https://www.ncbi.nlm.nih.gov/protein/YP_095692.1) | hypothetical protein lpg1665 |
| lpg1666 | 1849247 | 1850650 | - | [YP_095693.1](https://www.ncbi.nlm.nih.gov/protein/YP_095693.1) | hypothetical protein lpg1666 |
| lpg1673 | 1860352 | 1861671 | - | [YP_095700.1](https://www.ncbi.nlm.nih.gov/protein/YP_095700.1) | phosphoribosylamine-glycine ligase |
| lpg1680 | 1870228 | 1871607 | dsbD | [YP_095707.1](https://www.ncbi.nlm.nih.gov/protein/YP_095707.1) | thiol:disulfide interchange protein DsbD |
| lpg1690 | 1881178 | 1883853 | acnA | [YP_095717.1](https://www.ncbi.nlm.nih.gov/protein/YP_095717.1) | aconitate hydratase |
| lpg1696 | 1888202 | 1891366 | putA | [YP_095723.1](https://www.ncbi.nlm.nih.gov/protein/YP_095723.1) | bifunctional proline dehydrogenase/  pyrroline-5-carboxylate dehydrogenase |
| lpg1703 | 1898621 | 1899916 | - | [YP_095730.1](https://www.ncbi.nlm.nih.gov/protein/YP_095730.1) | SOS mutagenesis and repair UmuC protein |
| lpg1707 | 1902841 | 1904331 | + | [YP_095734.1](https://www.ncbi.nlm.nih.gov/protein/YP_095734.1) | succinylglutamic semialdehyde dehydrogenase |
| lpg1708 | 1904337 | 1905683 | astB | [YP_095735.1](https://www.ncbi.nlm.nih.gov/protein/YP_095735.1) | succinylarginine dihydrolase |
| lpg1814 | 2031718 | 2032272 | - | [YP_095840.1](https://www.ncbi.nlm.nih.gov/protein/YP_095840.1) | hypothetical protein lpg1814 |
| lpg1816 | 2033353 | 2034642 | - | [YP_095842.1](https://www.ncbi.nlm.nih.gov/protein/YP_095842.1) | major facilitator family transporter |
| lpg1817 | 2034644 | 2035651 | - | [YP_095843.1](https://www.ncbi.nlm.nih.gov/protein/YP_095843.1) | hypothetical protein lpg1817 |
| lpg1819 | 2036567 | 2038360 | - | [YP_095845.1](https://www.ncbi.nlm.nih.gov/protein/YP_095845.1) | lipid ABC transporter permease/ATP-binding protein |
| ≈45504 bp | | | | | |
| lpg2585 | 2916109 | 2916840 | pcgL | [YP_096590.1](https://www.ncbi.nlm.nih.gov/protein/YP_096590.1) | hypothetical protein lpg2585 |
| lpg2586 | 2917042 | 2918136 | - | [YP_096591.1](https://www.ncbi.nlm.nih.gov/protein/YP_096591.1) | cysteine protease |
| lpg2587 | 2918357 | 2919907 | - | [YP_096592.1](https://www.ncbi.nlm.nih.gov/protein/YP_096592.1) | thermolabile hemolysin |
| lpg2589 | 2921870 | 2923663 | dacB | [YP_096594.1](https://www.ncbi.nlm.nih.gov/protein/YP_096594.1) | D-alanyl-D-alanine carboxypeptidase |
| lpg2590 | 2923755 | 2924546 | spoOJ | [YP_096595.1](https://www.ncbi.nlm.nih.gov/protein/YP_096595.1) | chromosome partitioning protein ParB |
| lpg2593 | 2926079 | 2927362 | - | [YP_096598.1](https://www.ncbi.nlm.nih.gov/protein/YP_096598.1) | 16S rRNA (cytosine(967)-C(5))-methyltransferase |
| lpg2594 | 2927359 | 2928303 | fmt | [YP_096599.1](https://www.ncbi.nlm.nih.gov/protein/YP_096599.1) | methionyl tRNA formyltransferase |
| lpg2595 | 2928300 | 2928812 | def | [YP_096600.1](https://www.ncbi.nlm.nih.gov/protein/YP_096600.1) | peptide deformylase |
| lpg2596 | 2928906 | 2929943 | - | [YP_096601.1](https://www.ncbi.nlm.nih.gov/protein/YP_096601.1) | signal peptide protein |
| lpg2597 | 2929940 | 2931025 | - | [YP_096602.1](https://www.ncbi.nlm.nih.gov/protein/YP_096602.1) | DNA processing protein DprA |
| lpg2598 | 2931036 | 2931452 | - | [YP_096603.1](https://www.ncbi.nlm.nih.gov/protein/YP_096603.1) | hypothetical protein lpg2598 |
| lpg2599 | 2931531 | 2933810 | topA | [YP_096604.1](https://www.ncbi.nlm.nih.gov/protein/YP_096604.1) | DNA topoisomerase I |
| lpg2602 | 2936592 | 2937014 | - | [YP_096607.1](https://www.ncbi.nlm.nih.gov/protein/YP_096607.1) | hypothetical protein lpg2602 |
| lpg2604 | 2938631 | 2939434 | - | [YP_096609.1](https://www.ncbi.nlm.nih.gov/protein/YP_096609.1) | hypothetical protein lpg2604 |
| lpg2606 | 2940021 | 2940887 | - | [YP_096611.1](https://www.ncbi.nlm.nih.gov/protein/YP_096611.1) | glutamine amidotransferase |
| lpg2607 | 2941026 | 2943062 | pepO | [YP_096612.1](https://www.ncbi.nlm.nih.gov/protein/YP_096612.1) | metallopeptidase PepO |
| lpg2608 | 2943206 | 2944120 | lpxC | [YP_096613.1](https://www.ncbi.nlm.nih.gov/protein/YP_096613.1) | UDP-3-O |
| lpg2609 | 2944368 | 2945564 | ftsZ | [YP_096614.1](https://www.ncbi.nlm.nih.gov/protein/YP_096614.1) | cell division protein FtsZ |
| lpg2610 | 2945759 | 2947021 | ftsA | [YP_096615.1](https://www.ncbi.nlm.nih.gov/protein/YP_096615.1) | ATP-binding cell division protein FtsA |
| lpg2615 | 2951164 | 2952348 | ftsW | [YP_096620.1](https://www.ncbi.nlm.nih.gov/protein/YP_096620.1) | cell division protein FtsW |
| lpg2616 | 2952345 | 2953688 | murD | [YP_096621.1](https://www.ncbi.nlm.nih.gov/protein/YP_096621.1) | UDP-N-acetylmuramoyl-L-alanyl-D-glutamate synthetase |
| lpg2617 | 2953702 | 2954820 | mraY | [YP_096622.1](https://www.ncbi.nlm.nih.gov/protein/YP_096622.1) | phospho-N-acetylmuramoyl-pentapeptide-transferase |
| lpg2618 | 2954902 | 2956287 | murF | [YP_096623.1](https://www.ncbi.nlm.nih.gov/protein/YP_096623.1) | UDP-N-acetylmuramoyl-tripeptide--D-alanyl-D-alanine ligase |
| lpg2620 | 2957264 | 2960758 | - | [YP_096625.1](https://www.ncbi.nlm.nih.gov/protein/YP_096625.1) | chromosome segregation protein SMC |
| lpg2621 | 2960933 | 2961613 | - | [YP_096626.1](https://www.ncbi.nlm.nih.gov/protein/YP_096626.1) | acid phosphatase, class B |
| ≈43388bp | | | | | |
| lpg2625 | 2964464 | 2967667 | carB | [YP_096630.1](https://www.ncbi.nlm.nih.gov/protein/YP_096630.1) | carbamoyl phosphate synthase, large subunit |
| lpg2627 | 2968179 | 2969360 | - | [YP_096632.1](https://www.ncbi.nlm.nih.gov/protein/YP_096632.1) | hypothetical protein lpg2627 |
| lpg2633 | 2974501 | 2974818 | - | [YP_096638.1](https://www.ncbi.nlm.nih.gov/protein/YP_096638.1) | hypothetical protein lpg2633 |
| lpg2635 | 2976210 | 2977781 | mviN | [YP_096640.1](https://www.ncbi.nlm.nih.gov/protein/YP_096640.1) | virulence factor MviN |
| lpg2639 | 2981818 | 2985420 | enhC | [YP_096644.1](https://www.ncbi.nlm.nih.gov/protein/YP_096644.1) | enhanced entry protein EnhC |
| lpg2641 | 2986010 | 2986732 | enhA | [YP_096646.1](https://www.ncbi.nlm.nih.gov/protein/YP_096646.1) | enhanced entry protein EnhA |
| lpg2645 | 2991885 | 2993741 | uvrC | [YP_096650.1](https://www.ncbi.nlm.nih.gov/protein/YP_096650.1) | excinuclease ABC subunit C |
| lpg2648 | 2995794 | 2996891 | cinA | [YP_096653.1](https://www.ncbi.nlm.nih.gov/protein/YP_096653.1) | competence damage inducible protein CinA |
| lpg2651 | 2998381 | 2998692 | rplU | [YP_096656.1](https://www.ncbi.nlm.nih.gov/protein/YP_096656.1) | 50S ribosomal protein L21 |
| lpg2655 | 3001753 | 3002868 | - | [YP_096660.1](https://www.ncbi.nlm.nih.gov/protein/YP_096660.1) | sensory box protein |
| lpg2657 | 3004193 | 3006448 | feoB | YP_096662.1 | ferrous iron transport protein B |
| lpg2658 | 3006445 | 3006672 | feoA | YP_096663.1 | ferrous iron transporter A |
| lpg2659 | 3006761 | 3007852 | - | YP_096664.1 | ATPase N2B (nucleotide (GTP) binding protein) |
