# Supplementary material for: Genome analysis of Legionella pneumophila ST23 from various countries reveals highly similar strains
Source: Life Sci Alliance. 2022 Mar 2;5(6):e202101117. doi: 10.26508/lsa.202101117 (PMC8899845; doi:10.26508/lsa.202101117)
Supplement: Supplementary file 8 [file LSA-2021-01117_TableS8.docx]

**Table S8.** List of the sequenced genomes submitted to the GenBank

| **Strain ID** | **Source** | **Town** | **Year isolation** | **Investigation context** | **Monoclonal subgroup** | **tot length contigs>500** | **coverage (x)** | **Accession number** |
| --- | --- | --- | --- | --- | --- | --- | --- | --- |
| 228C | clinical | Bresso | 2014 | CA-O | Philadelphia | 3450145 | 107 | SAMN14088696 |
| 2251B | environmental | Bresso | 2014 | CA-O | France Allentown | 3403185 | 350 | SAMN14088697 |
| 2251C | environmental | Bresso | 2014 | CA-O | France Allentown | 3402505 | 370 | SAMN14088698 |
| 2251D | environmental | Bresso | 2014 | CA-O | France Allentown | 3402078 | 448 | SAMN14088699 |
| 427C | clinical | Bresso | 2018 | CA-O | Philadelphia | 3402733 | 447 | SAMN14088700 |
| 428C | clinical | Bresso | 2018 | CA-O | Philadelphia | 3403796 | 215 | SAMN14088701 |
| 435C | clinical | Bresso | 2018 | CA-O | Philadelphia | 3403828 | 206 | SAMN14088702 |
| 436C | clinical | Bresso | 2018 | CA-O | Philadelphia | 3403131 | 166 | SAMN14088703 |
| 2227A | environmental | Bresso | 2018 | CA-O | France Allentown | 3402791 | 299 | SAMN14088704 |
| 2252A | environmental | Bresso | 2018 | CA-O | Benidorm | 3376044 | 416 | SAMN14088705 |
| 2252C | environmental | Bresso | 2018 | CA-O | Benidorm | 3376872 | 454 | SAMN14088706 |
| 2253A | environmental | Bresso | 2018 | CA-O | Benidorm | 3376848 | 218 | SAMN14088707 |
| 2253B | environmental | Bresso | 2018 | CA-O | Benidorm | 3376435 | 405 | SAMN14088708 |
| 2253C | environmental | Bresso | 2018 | CA-O | Benidorm | 3376643 | 362 | SAMN14088709 |
| 2255A | environmental | Bresso | 2018 | CA-O | France Allentown | 3400413 | 173 | SAMN14088710 |
| 2256A | environmental | Bresso | 2018 | CA-O | France Allentown | 3372874 | 244 | SAMN14088711 |
| 2257A | environmental | Bresso | 2018 | CA-O | France Allentown | 3401957 | 279 | SAMN14088712 |
| 2258A | environmental | Bresso | 2018 | CA-O | France Allentown | 3402355 | 179 | SAMN14088713 |
| 2259A | environmental | Bresso | 2018 | CA-O | France Allentown | 3400906 | 341 | SAMN14088714 |
| 2260A | environmental | Bresso | 2018 | CA-O | France Allentown | 3403207 | 146 | SAMN14088715 |
| 2261A | environmental | Bresso | 2018 | CA-O | France Allentown | 3402460 | 257 | SAMN14088716 |
| 2452B1 | environmental | Bresso | 2018 | CA-O | Philadelphia | 3407389 | 87 | SAMN14088717 |
| 2452B2 | environmental | Bresso | 2018 | CA-O | Philadelphia | 3410679 | 202 | SAMN14088718 |
| 2452C1 | environmental | Bresso | 2018 | CA-O | Philadelphia | 3438995 | 103 | SAMN14088719 |
| 2452C2 | environmental | Bresso | 2018 | CA-O | Philadelphia | 3414629 | 170 | SAMN14088720 |
| 2452D1 | environmental | Bresso | 2018 | CA-O | Philadelphia | 3441276 | 158 | SAMN14088721 |
| 2452D2 | environmental | Bresso | 2018 | CA-O | Philadelphia | 3409474 | 196 | SAMN14088722 |
| 2452A | environmental | Bresso | 2018 | CA-O | Philadelphia | 3410292 | 141 | SAMN14088723 |
| 3699 | clinical | Roma | 2003 | CA-O | Philadelphia | 3397299 | 282 | SAMN14088724 |
| 3712 | environmental | Roma | 2003 | CA-O | Philadelphia | 3396991 | 192 | SAMN14088725 |
| 3718 | environmental | Roma | 2003 | CA-O | Philadelphia | 3397857 | 131 | SAMN14088726 |
| 3713 | environmental | Roma | 2003 | CA-O | Philadelphia | 3396656 | 101 | SAMN14088727 |
| 3777 | environmental | Roma | 2003 | CA-O | Philadelphia | 3397318 | 331 | SAMN14088728 |
| 4454 | clinical | Cesano Maderno | 2007 | CA-O | Knoxville | 3401224 | 249 | SAMN14088729 |
| 18C | clinical | Cesano Maderno | 2008 | CA-O | Philadelphia | 3401116 | 306 | SAMN14088730 |
| 4407 | environmental | Cesano Maderno | 2007 | CA-O | Knoxville | 3402261 | 230 | SAMN14088731 |
| 22A | environmental | Cesano Maderno | 2007 | CA-O | Knoxville | 3511864 | 28 | SAMN14088732 |
| 11A | environmental | Cesano Maderno | 2007 | CA-O | Philadelphia | 3400281 | 113 | SAMN14088733 |
| 151A | environmental | Cesano Maderno | 2007 | CA-O | Knoxville | 3401326 | 301 | SAMN14088734 |
| 160A | environmental | Cesano Maderno | 2008 | CA-O | Philadelphia | 3400415 | 234 | SAMN14088735 |
| 143C | clinical | Lazise | 2011 | TA-O | France Allentown Philadelphia | 3462464 | 252 | SAMN14088736 |
| 594A | environmental | Lazise | 2011 | TA-O | France Allentown Philadelphia | 3460555 | 83 | SAMN14088737 |
| 595A | environmental | Lazise | 2011 | TA-O | France Allentown Philadelphia | 3462727 | 173 | SAMN14088738 |
| 2417 | clinical | Monza | 1995 | Unknown-SP | Philadelphia | 3497893 | 76 | SAMN14088739 |
| 2418 | clinical | Monza | 1995 | Unknown-SP | Philadelphia | 3455212 | 76 | SAMN14088740 |
| 3933 | clinical | Trento | 2004 | CA | Philadelphia | 3496297 | 39 | SAMN14088741 |
| 1C | clinical | Roma | 2007 | CA-SP | Knoxville | 3429895 | 87 | SAMN14088742 |
| 299C | clinical | Verona | 2011 | Unknown-SP | Philadelphia | 3442583 | 78 | SAMN14088743 |
| 300C | clinical | Verona | 2011 | Unknown-SP | Philadelphia | 3434430 | 218 | SAMN14088744 |
| 717A | environmental | Ravenna | 2012 | TA-O | France Allentown Philadelphia | 3479113 | 170 | SAMN14088745 |
| 181C | clinical | Ravenna | 2012 | TA-O | France Allentown Philadelphia | 3471313 | 94 | SAMN14088746 |
| 1214A | environmental | Piacenza | 2015 | CA-O | Camperdown | 3559520 | 69 | SAMN14088747 |
| 323C | clinical | Cesena | 2016 | CA-SP | Philadelphia | 3415541 | 157 | SAMN14088748 |
| 325C | clinical | Bolzano | 2017 | TA-SP | Philadelphia | 3410269 | 168 | SAMN14088749 |
| 383C | clinical | Ancona | 2017 | Unknown-SP | Philadelphia | 3418968 | 71 | SAMN14088750 |
| 384C | clinical | Como | 2017 | CA-O | Philadelphia | 3404610 | 272 | SAMN14088751 |
| 415C | clinical | Milano | 2017 | CA-SP | Philadelphia | 3474128 | 132 | SAMN14088752 |
| 419C | clinical | Bolzano | 2017 | CA-SP | Philadelphia | 3479656 | 193 | SAMN14088753 |
| 1762A | environmental | Como | 2017 | CA-O | Philadelphia | 3406506 | 186 | SAMN14088754 |
| 472C | clinical | Milano | 2018 | CA-O | Philadelphia | 3511869 | 211 | SAMN14088755 |
| 483C | clinical | Mantova | 2018 | CA-O | France Allentown | 3310574 | 109 | SAMN14088756 |
| 2301A | environmental | Brescia | 2018 | CA-O | France Allentown | 3506629 | 190 | SAMN14088757 |

CA-O= Community Acquired - Outbreak; TA-O= Travel Associated – Outbreak; CA-SP= Community Acquired Sporadic; TA-SP Travel Associated Sporadic; SP= Sporadic
