# Supplementary material for: Genome analysis of Legionella pneumophila ST23 from various countries reveals highly similar strains
Source: Life Sci Alliance. 2022 Mar 2;5(6):e202101117. doi: 10.26508/lsa.202101117 (PMC8899845; doi:10.26508/lsa.202101117)
Supplement: Supplementary file 10 [file LSA-2021-01117_TableS10.docx]

| **Table S10. ST23 from other countries and other STs used in this study** | | | | | | |  |  |  |
| --- | --- | --- | --- | --- | --- | --- | --- | --- | --- |
| **Isolate name** | **ST** | **Sg** | **Source** | **Country** | **Year** | **Accession number/** | | | |
|  |  |  |  |  |  | **Reference** | | | |
| DK01 | 23 | 1 | clin | Capital Region DK | 2019 | This study | | | |
| DK02 | 23 | 1 | clin | Southern Denmark | 2017 | This study | | | |
| DK03 | 23 | 1 | clin | Southern Denmark | 2017 | This study | | | |
| DK04 | 23 | 1 | clin | Capital Region DK | 2017 | This study | | | |
| DK05 | 23 | 1 | clin | Capital Region DK | 2017 | This study | | | |
| DK06 | 23 | 1 | clin | Berlin/Germany | 2013 | This study | | | |
| DK07 | 23 | 1 | clin | Southern Denmark | 2012 | This study | | | |
| DK08 | 23 | 1 | clin | Capital Region DK | 2011 | This study | | | |
| DK09 | 23 | 1 | clin | Central DK Region | 2010 | This study | | | |
| DK10 | 23 | 1 | clin | Region Zealand | 2009 | This study | | | |
| DK11 | 23 | 1 | clin | North DK Region | 2009 | This study | | | |
| 4599 | 23 | 1 | clin | Southern Europe | 2003 | ERR3219065 | | | |
| 4606 | 23 | 1 | env | Southern Europe | 2003 | ERR3219070 | | | |
| 4697 | 23 | 1 | clin | Southern Europe | 2008 | ERR3219120 | | | |
| 4711 | 23 | 1 | clin | Southern Europe | 2008 | ERR3219128 | | | |
| 4782 | 23 | 1 | clin | Southern Europe | 2010 | ERR3219140 | | | |
| 4787 | 23 | 1 | clin | Southern Europe | 2011 | ERR3219145 | | | |
| 4440 | 23 | 1 | clin | Southern Europe | 1999 | ERR3218978 | | | |
| EUL8 | 23 | 1 | clin | Switzerland | 1993 | ERR376633 | | | |
| EUL11 | 23 | 1 | env | Switzerland | 1993 | ERR376636 | | | |
| EUL12 | 23 | 1 | env | Switzerland | 1993 | ERR376637 | | | |
| EUL41 | 23 | 1 | clin | Italy | 1999 | ERR376666 | | | |
| EUL4 | 23 | 1 | clin | Switzerland | 1991 | ERR376721 | | | |
| EUL28 | 23 | 1 | clin | France | 1994 | ERR376722 | | | |
| EUL129 | 23 | 1 | clin | Croatia | 1987 | ERR376762 | | | |
| EUL130 | 23 | 1 | clin | Croatia | 1987 | ERR376703 | | | |
| H063280001 | 23 | 1 | clin | Uk | 2006 | ERR315663 | | | |
| LG 0834 5006 | 23 | 1 | clin | France | 2008 | David *et al.* (2016) | | | |
| LG 0751 2008 | 23 | 1 | clin | France | 2007 | David *et al.* (2016) | | | |
| LG 0839 2025 | 23 | 1 | clin | France | 2008 | David *et al.* (2016) | | | |
| HL 0709 3017 | 23 | 1 | clin | France | 2007 | David *et al.* (2016) | | | |
| HL 0307 1012 | 23 | 1 | clin | France | 2003 | David *et al.* (2016) | | | |
| HL 0339 3028 | 23 | 1 | clin | France | 2003 | David *et al.* (2016) | | | |
| HL 0437 1017 | 23 | 1 | clin | France | 2004 | David *et al.* (2016) | | | |
| HL 0443 3031 | 23 | 1 | clin | France | 2004 | David *et al.* (2016) | | | |
| HL 0506 3005 | 23 | 1 | clin | France | 2005 | David *et al.* (2016) | | | |
| HL 0532 2037 | 23 | 1 | env | France | 2005 | David *et al.* (2016) | | | |
| HL 0541 5018 | 23 | 1 | clin | France | 2005 | David *et al.* (2016) | | | |
| HL 0604 3045 | 23 | 1 | clin | France | 2006 | David *et al.* (2016) | | | |
| HL 0637 3021 | 23 | 1 | clin | France | 2006 | David *et al.* (2016) | | | |
| LG08345006 | 23 | 1 | clin | France | 2008 | David *et al.* (2016) | | | |
| EUL88 | 1 | 1 | clin | Denmark | 1995 | ERR332174 | | | |
| EUL93 | 1 | 1 | clin | Denmark | 1992 | ERR332179 | | | |
| EUL10 | 1 | 1 | env | Switzerland | 1989 | ERR376635 | | | |
| EUL1 | 1 | 1 | clin | Switzerland | 1998 | ERR376626 | | | |
| EUL21 | 1 | 1 | env | UK | 1999 | ERR376638 | | | |
| LC5694 | 37 | 1 | clin | UK | 2000 | ERR363891 | | | |
| LC5722 | 37 | 1 | clin | UK | 2000 | ERR363892 | | | |
| LC5755 | 37 | 1 | clin | UK | 2000 | ERR363894 | | | |
| H120240685 | 37 | 1 | clin | Slovenia | 2010 | ERR363992 | | | |
| H114100406 | 62 | 1 | clin | Greece | 2011 | ERR364016 | | | |
| H120240362 | 62 | 1 | clin | UK | 2012 | ERR364025 | | | |
| H104640262 | 62 | 1 | clin | Uk | 2010 | ERR364019 | | | |
| H123140428 | 62 | 1 | env | UK | 2012 | ERR364015 | | | |
| H123460520 | 62 | 1 | clin | UK | 2012 | ERR364014 | | | |
