# Supplementary material for: Genome analysis of Legionella pneumophila ST23 from various countries reveals highly similar strains
Source: Life Sci Alliance. 2022 Mar 2;5(6):e202101117. doi: 10.26508/lsa.202101117 (PMC8899845; doi:10.26508/lsa.202101117)
Supplement: Supplementary file 11 [file LSA-2021-01117_TableS11.docx]

| **Table S11. Genomes with SNPs ranging 1 to 40** | | | |
| --- | --- | --- | --- |
| **Strain ID** | **Source** | **Origin** | **Year isolation** |
|  |  |  |  |
| 383C | clinical | Ancona | 2017 |
| 419C | clinical | Bolzano | 2017 |
| 2301A | environmental | Brescia | 2018 |
| 228C | clinical | Bresso | 2014 |
| 2251B | environmental | Bresso | 2014 |
| 2251C | environmental | Bresso | 2014 |
| 2251D | environmental | Bresso | 2014 |
| 2452B1 | environmental | Bresso | 2018 |
| 2452B2 | environmental | Bresso | 2018 |
| 2452C1 | environmental | Bresso | 2018 |
| 2255A | environmental | Bresso | 2018 |
| 2258A | environmental | Bresso | 2018 |
| 427C | clinical | Bresso | 2018 |
| 428C | clinical | Bresso | 2018 |
| 435C | clinical | Bresso | 2018 |
| 436C | clinical | Bresso | 2018 |
| 2227A | environmental | Bresso | 2018 |
| 2256A | environmental | Bresso | 2018 |
| 2257A | environmental | Bresso | 2018 |
| 2259A | environmental | Bresso | 2018 |
| 2260A | environmental | Bresso | 2018 |
| 2261A | environmental | Bresso | 2018 |
| 2452C2 | environmental | Bresso | 2018 |
| 2452D1 | environmental | Bresso | 2018 |
| 2452D2 | environmental | Bresso | 2018 |
| 2452A | environmental | Bresso | 2018 |
| 2252A | environmental | Bresso | 2018 |
| 143C | clinical | Lazise | 2011 |
| 594A | environmental | Lazise | 2011 |
| 595A | environmental | Lazise | 2011 |
| 483C | clinical | Mantova | 2018 |
| 472C | clinical | Milano | 2018 |
| 2418 | clinical | Monza | 1995 |
| 1214A | environmental | Piacenza | 2015 |
| 181C | clinical | Ravenna | 2012 |
| 717A | environmental | Ravenna | 2012 |
| 3699 | clinical | Rome | 2003 |
| 3712 | environmental | Rome | 2003 |
| 3718 | environmental | Rome | 2003 |
| 3713 | environmental | Rome | 2003 |
| 3777 | environmental | Rome | 2003 |
| EUL00008 | clinical | Switzerland | 1993 |
| EUL00011 | environmental | Switzerland | 1993 |
|  |  |  |  |
