# Supplementary material for: Genome analysis of Legionella pneumophila ST23 from various countries reveals highly similar strains
Source: Life Sci Alliance. 2022 Mar 2;5(6):e202101117. doi: 10.26508/lsa.202101117 (PMC8899845; doi:10.26508/lsa.202101117)
Supplement: Supplementary file 12 [file LSA-2021-01117_TableS12.docx]

| **Table S12. Genomes with SNPs ranging 171 to 802** | | | |  |
| --- | --- | --- | --- | --- |
| **Strain ID** | **Source** | **Origin** | **Year isolation** | |
|  |  |  |  |  |
| 325C | clinical | Bolzano | 2017 | |
| 2252A | environmental | Bresso | 2018 | |
| 2252C | environmental | Bresso | 2018 | |
| 2253A | environmental | Bresso | 2018 | |
| 2253B | environmental | Bresso | 2018 | |
| 2253C | environmental | Bresso | 2018 | |
| 4454 | clinical | Cesano Maderno | 2007 | |
| 4407 | environmental | Cesano Maderno | 2007 | |
| 22A | environmental | Cesano Maderno | 2007 | |
| 11A | environmental | Cesano Maderno | 2007 | |
| 151A | environmental | Cesano Maderno | 2007 | |
| 18C | clinical | Cesano Maderno | 2008 | |
| 160A | environmental | Cesano Maderno | 2008 | |
| 323C | clinical | Cesena | 2016 | |
| 384C | clinical | Como | 2017 | |
| 1762A | environmental | Como | 2017 | |
| 415C | clinical | Milano | 2017 | |
| 1C | clinical | Roma | 2007 | |
| 300C | clinical | Verona | 2011 | |
| EUL00012 | environmental | Switzerland | 1993 | |
